# Transcriptomic analysis of differentially expressed genes during anther development in genetic male sterile and wild type cotton by digital gene-expression profiling

**DOI:** 10.1186/1471-2164-14-97

**Published:** 2013-02-12

**Authors:** Mingming Wei, Meizhen Song, Shuli Fan, Shuxun Yu

**Affiliations:** 1 College of Agriculture, Northwest A&F University, 712100, Yangling, Shaanxi, P. R. China; 2 State Key Laboratory of Cotton Biology, Institute of Cotton Research of CAAS, P. R. Chinese Academy of Agriculture Sciences (CAAS), 455000, Anyang, Henan, P. R. China

## Abstract

**Background:**

Cotton (*Gossypium hirsutum*) anther development involves a diverse range of gene interactions between sporophytic and gametophytic tissues. However, only a small number of genes are known to be specifically involved in this developmental process and the molecular mechanism of the genetic male sterility (GMS) is still poorly understand. To fully explore the global gene expression during cotton anther development and identify genes related to male sterility, a digital gene expression (DGE) analysis was adopted.

**Results:**

Six DGE libraries were constructed from the cotton anthers of the wild type (WT) and GMS mutant (in the WT background) in three stages of anther development, resulting in 21,503 to 37,352 genes detected in WT and GMS mutant anthers. Compared with the fertile isogenic WT, 9,595 (30% of the expressed genes), 10,407 (25%), and 3,139 (10%) genes were differentially expressed at the meiosis, tetrad, and uninucleate microspore stages of GMS mutant anthers, respectively. Using both DGE experiments and real-time quantitative RT-PCR, the expression of many key genes required for anther development were suppressed in the meiosis stage and the uninucleate microspore stage in anthers of the mutant, but these genes were activated in the tetrad stage of anthers in the mutant. These genes were associated predominantly with hormone synthesis, sucrose and starch metabolism, the pentose phosphate pathway, glycolysis, flavonoid metabolism, and histone protein synthesis. In addition, several genes that participate in DNA methylation, cell wall loosening, programmed cell death, and reactive oxygen species generation/scavenging were activated during the three anther developmental stages in the mutant.

**Conclusions:**

Compared to the same anther developmental stage of the WT, many key genes involved in various aspects of anther development show a reverse gene expression pattern in the GMS mutant, which indicates that diverse gene regulation pathways are involved in the GMS mutant anther development. These findings provide the first insights into the mechanism that leads to genetic male sterility in cotton and contributes to a better understanding of the regulatory network involved in anther development in cotton.

## Background

Male sterility is a simple and efficient pollination control system that is widely exploited in hybrid breeding. In cotton breeding, cytoplasmic male sterility (CMS) and genetic male sterility (GMS) have been used to produce hybrid seeds. Both types of lines have a maternally (former) or nuclear (later) inherited trait and each line is unable to produce or release functional pollen. Such lines are suitable as maternal plants for the utilization of hybrid vigor.

Although the CMS system can economically generate a completely male-sterile population, hybrid seed production using this system involves development of three lines, it usually takes years to develop the maintainer and restorer lines because most CMS systems have stringent restoring-maintaining relationships, and any CMS system potentially causes negative cytoplasmic effects and shows unstable sterility [1]. The advantage of GMS system is that it involves only two lines and the genes responsible for male sterility are relatively easy to transfer to any desired genetic background. For example, in genetic male sterility controlled by a recessive gene(s) (RGMS), most breeding lines can serve as restorers, thus it is easy to combine elite lines to produce hybrids that show high heterosis. The molecular mechanism of GMS is currently a research hotspot in plant science.

GMS-sterile lines are mainly controlled by alleles of nuclear genes designated as ‘ms’ that affect male reproduction. These alleles are usually recessive, but some are dominant (Ms) and both types are typically expressed in specific sporophytic tissues at different stages. In *G*. *hirsutum*, the GMS mutant in ‘Dong A’ is controlled by one pair of recessive genes [2], and it has the same genetic background with its wild type (WT). Therefore, they are ideal genetic materials for studying cotton anther development and male sterility.

In plants, anther is a bilaterally symmetrical structure with four lobes, each lobe containing meiotic cells at the center surrounded by four somatic cell layers, which are the epidermis, the endothecium, the middle layer, and the tapetum from the surface to the interior [3]. The meiotic cells produce microspore in anther lodes. Among the four somatic cell layers, the tapetum cells are of considerable physiological significance because all nutritional materials entering the sporogenous cells passes through or originates from the tapetum [4]. Tapetal aberrations are frequently observed in male sterile mutants, with premature or delayed degradation of the tapetum resulting in male sterility [5].

The anther formation and maturation is a critical phase in the plant life cycle, which commences at the end of meiosis with the formation of a tetrad and ends at the dehiscence of anthers when the mature pollen grains are released. This process involves a diverse range of gene interactions between sporophytic and gametophytic tissues [6,7]. Using forward and reverse genetic approaches, a growing number of genes have been identified to have vital roles in anther development [8]. For instance, SPOROCYTELESS (SPL)/NOZZLE (NZZ) gene required for cell division and differentiation is essential for the earliest anther development in *Arabidopsis*[9,10]. ABORTED MICROSPORES (AMS) is involved in tapetal development and microspore development in rice and *Arabidopsis*[11]. TAPETAL DETERMINANT1 (TPD1), DYSFUNCTIONAL TAPETUM (DYT1), and UNDEVELOPED TAPETUM1 are involved in tapetal and microsporocyte determination [12-14]. MALE STERILITY1 (MS1) is required for tapetal development and pollen wall formation in *Arabidopsis*[15]. MALE STERILITY2 (MS2) is involved in pollen wall biosynthesis in rice [16]. Cytochrome P450 family member CYP704B1 and CYP704B2 are essential for the formation of both cuticle and extine during plant male reproductive and spore development in *Arabidopsis* and rice [17,18]. LAP5 and LAP6 encode anther-specific proteins with similarity to chalcone synthase essential for pollen extine development in Arabidopsis [19]. However, the regulatory mechanism underlying anther development as well as the genetic network that governs male sterility is yet not to be fully understood.

In recent decades, most studies on male sterility focused on differential display methods, such as amplified fragment length polymorphisms [20,21]. These low-throughput methods are inadequate to fully detect gene expression in different samples. Although transcriptome profiling studies based on microarray data can detect detailed gene expression in different samples, this approach also has limitations because genes are represented by unspecific probe sets and cannot be reliably detected at low expression levels. High-throughput tag-sequencing for digital gene expression (DGE) analysis is a powerful, recently developed tool that allows the concomitant sequencing of millions of signatures to the genome, and identification of specific genes and the abundance of gene expression in a sample tissue [22]. This method provides a more qualitative and quantitative description of gene expression than previous microarray-based assays [23]. In a direct comparison with high-throughput mRNA sequencing (RNA-seq), both methods provide similar assessments of relative transcript abundance, but DGE better detects expression differences for poorly expressed genes and does not exhibit transcript length bias [24].

In the present study, DGE based on the Solexa Genome Analyzer platform was applied to analyze the complex regulatory network underlying anther development and to investigate the differences in gene expression between the ‘Dong A’ male sterile mutant and its wild type (WT). After high-throughput tag-sequencing, an integrated bioinformatic analysis was performed to identify the expression patterns of genes and critical pathways in the male-sterile mutant and WT at three stages of male gametophyte development. The aim of the study was to gain increased insight into the molecular mechanisms of cotton male sterility. The results yielded sets of up-regulated and down-regulated genes associated with male sterility, and candidate genes associated with cotton male sterility are discussed. Comparison of the gene expression patterns at three developmental stages of the WT and GMS mutant anther provides an improved understanding of the molecular mechanisms of cotton anther development and GMS.

## Results

### Phenotypic analysis of impaired anthers in cotton male-sterile mutant

**Figure 1:**
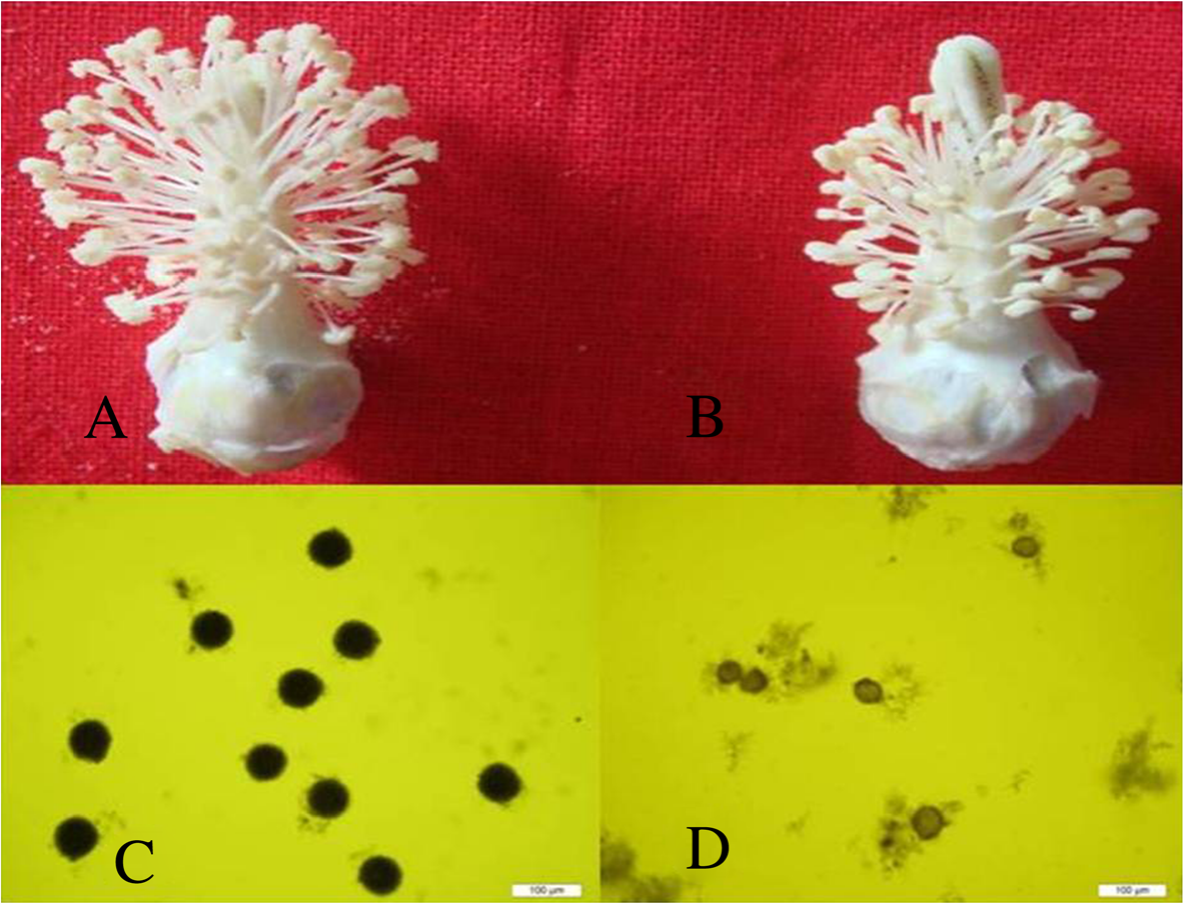
**Flowers and pollen grains of the wild type (WT) and GMS mutant.** Floral phenotypes of *G*. *hirsutum* ‘Dong A’ (WT: **A**) and GMS mutant of ‘Dong A’ (WT: **B**). Pollen grains stained with 2% I_2_-KI at 0 DPA of the WT (**C**) and the GMS mutant anther (**D**). Scale bar = 100 μm.

At one day post anthesis (DPA), *G*. *hirsutum* ‘Dong A’ (WT) showed normal floral phenotypes (Figure 1A), whereas the male sterile mutant (GMS mutant) showed abnormal floral phenotypes (Figure 1B) with shorter stima and filament. Pollen grains in the WT and GMS mutant were stained with 2% I_2_-KI to detect starch activity at 0 DPA. Many pollen grains were deeply stained in the WT (Figure 1C), but no pollen grains were stained in the GMS mutant (Figure 1D), which indicated that little nutritional materials were accumulated in the GMS mutant microspores.

**Figure 2:**
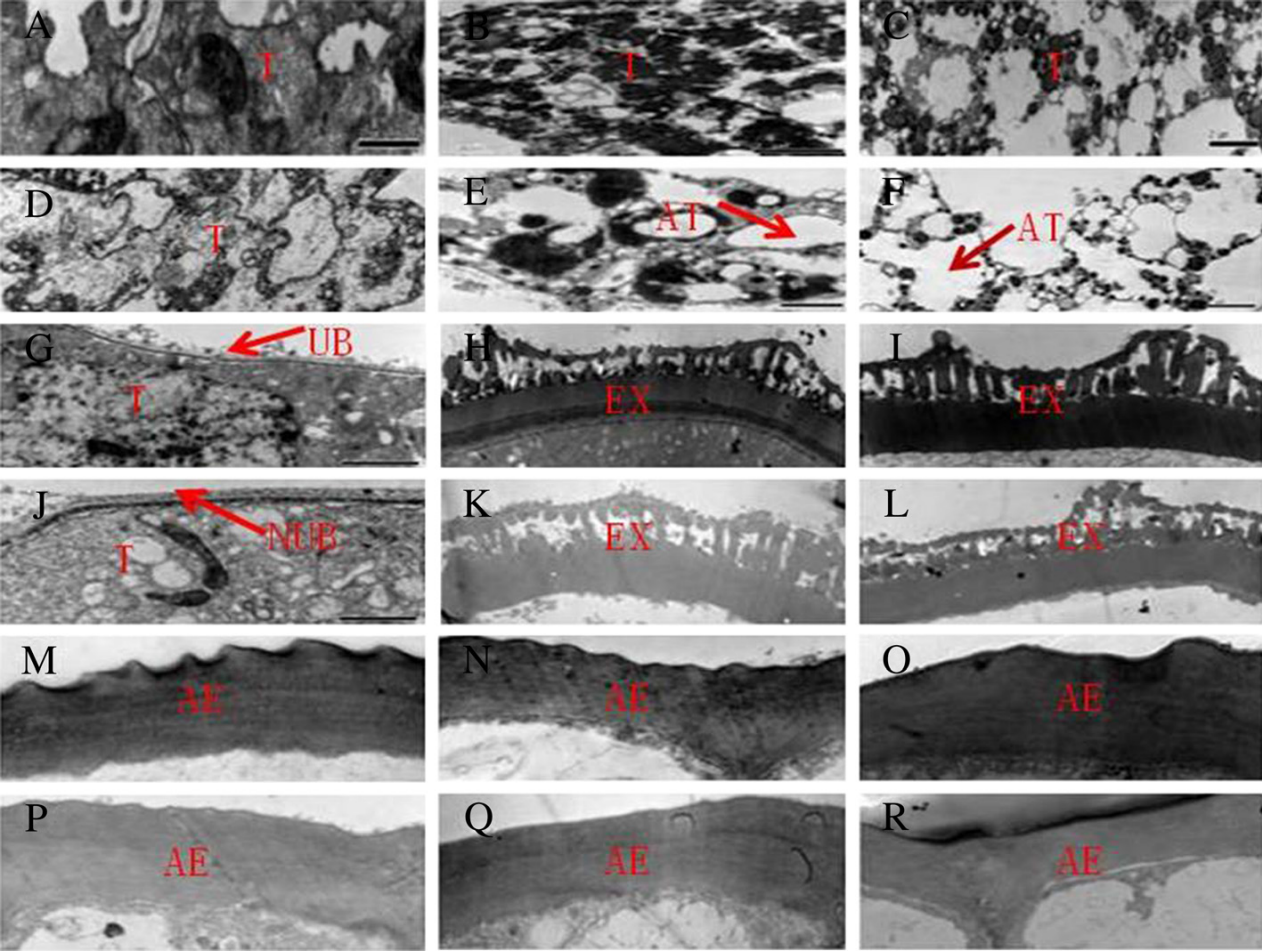
**TEM analysis of anthers in the WT and GMS mutant.** Transverse sections of the (**A-C**) WT and (**D-F**) GMS mutant tapetum at the (**A, D**) meiosis, (**B, E**) tetrad, and (**C, F**) uninucleate microspore stages. Extine development in (**G-I**) WT and (**J-L**) GMS mutant microspores at the (**G, J**) meiosis, (**H, K**) tetrad, and (**I, L**) uninucleate microspore stages. Outer wall of anther epidermal cells in the (**M-O**) WT and (**P-R**) GMS mutant at the (**M, P**) meiosis, (**N, Q**) tetrad, and (**O, R**) uninucleate microspore stages. T: Tapetal layer; AT: abnormal tapetal layer; UB: Ubisch body; NUB: no Ubisch body; Ex: extine; AE: anther epidermal cuticle. Bars = 2 μm (**A-L**), 1 μm (**M-R**).

To gain more detailed insights into the cellular defects during pollen development in the GMS mutant, anther samples from the WT and GMS mutant were examined by transmission electron microscopy (TEM) at different stages of development (Figure 2). The results of TEM observation showed that the tapetal cells of WT anthers were strongly stained during the meiosis stage, and some Ubisch bodies were extruded to the locular side of the tapetum (Figure 2A and G). At the tetrad stage, the tapetal cells appeared slightly vacuolated, and initial primexine formation was observed on the microspore surface. In addition, thick, protrudent, hair-like structures (probaculae) were observed outside the plasma membrane (Figure 2B and H), which indicated that sporopollenin components were actively transported from the tapetum to the microspores. This trend was further enhanced at the uninucleate microspore stage of WT anthers, in which the extine was thicker and stained strongly, and columnar baculae in the extine were observed (Figure 2I). This observation indicated that additional sporopollenin was deposited on the surface of the microspore. Microspore development in the GMS mutant was similar to that of the WT, but the tapetal layer in the GMS mutant stained less intensely and was more vacuolated, which indicated that the tapetal cells in the GMS mutant underwent acute and abnormal degradation (Figure 2D, E, and F). Furthermore, no Ubisch bodies were observed outside the tapetal layer of the GMS mutant (Figure 2J) and only a thin extine layer developed (Figure 2K and L), which indicated that sporopollenin synthesis was deficient in the GMS mutant.

From meiosis to the uninucleate microspore stage, the anther epidermal cell walls of the WT were strongly stained and gradually thickened, and decreased in electron density; the anther cuticle also became thicker (Figure 2M, N and O). Surprisingly, although the epidermis of the GMS mutant anther developed a cell wall, the wall was weakly stained, which indicated that decreased amounts of lipophilic materials were deposited in the outer epidermal cell wall. Moreover, the thickness of the cell wall and cuticle were greatly reduced in the GMS mutant (Figure 2P, Q and R). Thus, abnormal synthesis of the pollen wall and defective formation of the anther epidermal cell wall and cuticle in the GMS mutant anthers were indicated.

### Analysis of digital gene expression (DGE) libraries

Previous studies have demonstrated that the peak of male sterility mainly occurs in the uninucleate microspore stage of anthers in ‘Dong A’ GMS mutant [25]. Therefore, not only the uninucleate microspore stage anthers, but also anthers before the uninucleate microspore stage should be collected to analyze the differentially expressed genes between the WT and the GMS mutant anther. Based on this fact, anthers were harvested at the meiosis stage (WT: F-1; mutant: S-1), tetrad stage (WT: F-2; mutant: S-2), and uninucleate microspore stage (WT: F-3; mutant: S-3) to construct six DGE libraries. The Solexa Genome Analyzer platform was used to perform high-throughput tag-sequencing analysis of cotton anther libraries. The transcriptome during cotton anther development was characterized and differences in the gene regulatory pathways were analyzed at the three stages of WT and GMS mutant anther development.

The six DGE libraries were sequenced and generated 5.6–5.9 million high-quality tags. The number of tag entities with unique nucleotide sequences ranged from 177,594 to 282,106 (Table 1). The number of unique distinct clean tag sequences ranged from 79,790 to 163,814 (Table 1). The F-2 library contained the highest number of distinct clean tags; the other four libraries contained similar numbers. In addition, the F-2 library showed the highest ratio of number of distinct clean tags to total number of clean tags, and the lowest percentage of distinct high copy number tags (Additional file 1). These data suggested that more genes were detected in the F-2 library than in the other five libraries, and more transcripts were expressed at lower levels in the F-2 library. In addition, more than 41.49–55.97% of the highly regulated genes were found to be orphan sequences, i.e. no homologues were identified in the National Center of Biotechnological Information databases. This result might indicate that many unique processes and pathways are involved in *G*. *hirsutum* anther development.

**Table 1:** Categorization and abundance of tags

**Summary**		**F-1**	**F-2**	**F-3**	**S-1**	**S-2**	**S-3**
Raw data	Total	6057743	6106800	5907794	5926547	5891017	6042253
	Distinct tags	466231	457518	389247	252111	421049	345292
Clean tags	Total number	5766711	5928700	5696273	5849242	5661517	5871546
	Distinct tag numbers	195469	282106	180025	181205	209697	177594
All tag mapping to gene	Total number	4418440	4590068	4300473	4207316	4201776	4060397
	Total % of clean tags	76.62%	77.42%	75.50%	71.93%	74.22%	69.15%
	Distinct tag numbers	106159	163814	105324	79790	105946	92188
	Distinct tag % of clean tags	54.31%	58.07%	58.51%	44.03%	50.52%	51.91%
Unambiguous Tag mapping to gene	Total number	2216772	2193183	2227360	1948213	2138430	2192477
	Total % of clean tags	38.44%	36.99%	39.10%	33.31%	37.77%	37.34%
	Distinct tag numbers	72551	111516	71585	51219	72232	63400
	Distinct Tag % of clean tags	37.12%	39.53%	39.76%	28.27%	34.45%	35.70%
All tag-mapped genes	Number	66081	77376	66172	56741	67267	62364
	% of ref genes	56.71%	66.41%	56.79%	48.70%	57.73%	53.52%
Unambiguous tag-mapped genes	Number	34451	42940	33937	26642	35072	31397
	% of ref genes	29.57%	36.85%	29.13%	22.86%	30.10%	26.95%
Unknown tags	Total number	1348271	1338632	1395800	1641926	1459741	1811149
	Total % of clean tags	23.38%	22.58%	24.50%	28.07%	25.78%	30.85%
	Distinct tag numbers	89310	118292	74701	101415	103751	85406
	Distinct tag % of clean tags	45.69%	41.93%	41.49%	55.97%	49.48%	48.09%

Heterogeneity and redundancy are two significant characteristics of mRNA expression. A small number of mRNAs are present at a very high abundance, whereas expression of the majority of mRNAs remains at a very low level. The distribution of clean tag expression can be used to evaluate the normality of the whole DGE data. In the present study, the distribution of the distinct clean tag copy numbers showed extremely similar tendencies. Among the distinct clean tags in the six libraries, only 3.2–5.2% possessed more than 100 copies. The majority of distinct clean tags (28.64–60.88%) had 2–5 copies (Additional file 1: Figure S1), which indicated that the whole DGE data among the six libraries was normally distributed.

### Analysis of tag mapping

A reference gene database that included 116, 520 sequences of the *G*. *hirsutum* unigenes was preprocessed for tag mapping (http://occams.dfci.harvard.edu/pub/bio/tgi/data/Gossypium). Among the reference sequences, the genes with a CATG site accounted for 87.52% of the total. To obtain the reference tags, all of the CATG +17 tags in the gene were taken as gene reference tags. Finally, 284,576 total reference tag sequences with 236,167 unambiguous reference tags were obtained in the six libraries. On this basis, 44,968 unambiguous tag-mapped genes were detected in the six DGE libraries.

To estimate whether the sequencing depth was sufficient for the transcriptome coverage, the sequencing saturation in the six libraries was analyzed. The genes that were mapped by all clean tags and unambiguous clean tags increased with the total number of tags. However, when the sequencing counts reached 2 million tags or higher, the number of detected genes was saturated (Additional file 2). Given that Solexa sequencing can distinguish transcripts that originate from both DNA strands, using the strand-specific nature of the sequencing tags obtained, we found that 26.25–33.96% distinct clean tags showed a perfect match to sense strand-specific transcripts, and 7.43–12.98% distinct clean tags showed a perfect match to antisense strand-specific transcripts in the six libraries (Additional file 3). These results indicated that antisense genes also play important roles in the transcriptional regulation of cotton anther development.

### Transcriptome diversity quantified by DGE profiles in the WT and GMS mutant anthers

**Figure 3:**
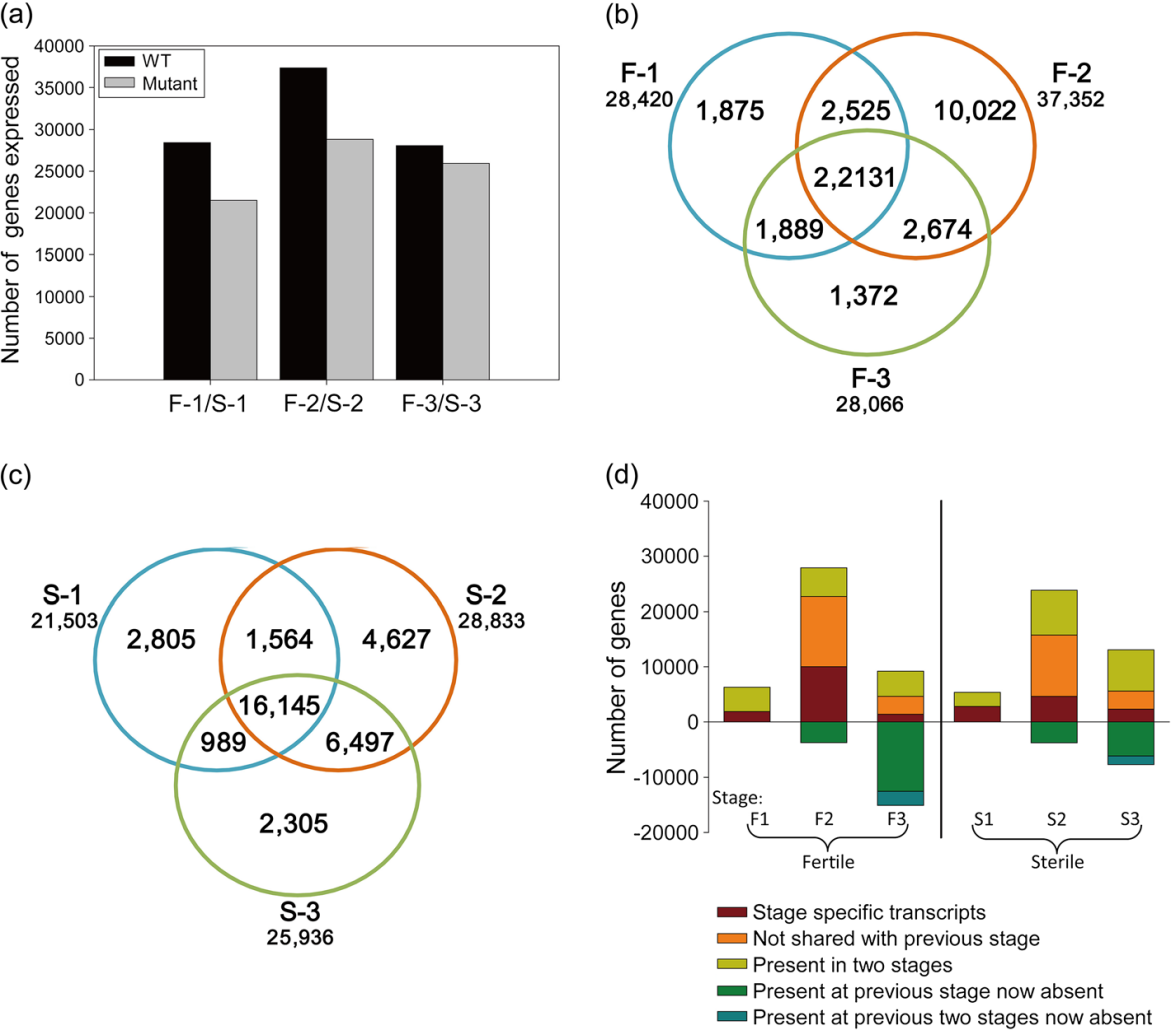
**Transcriptome analysis of the WT and GMS mutant anthers.** (**a**) Transcriptome sizes at three stages of the WT and GMS mutant anthers. (**b**), (**c**) Venn diagram showing the overlaps between anther stages of WT and GMS mutant (combined according to similarities in development). The number below each stage designation is the total transcripts detected in that stage(s). (**d**) Analysis of the progression of transcriptome changes during anther development of WT and GMS mutant anthers. The approximately 22,131 and 16,145 transcripts shared by three stages of WT and GMS mutant anthers are not shown, respectively. Numbers above the x-axis represent transcripts present in the indicated stage that are: stage specific (fufous); not present in the prior stage but shared with another stage (orange); or shared with the prior stage but missing in at least one other stage (yellow). Numbers below the x-axis represent transcripts present in the prior stage that are not detected in the current stage (from the category with green and blue color).

Based on deep sequencing of the six DGE libraries in the current study, 28,420 (24.4% of reference genes in cotton) and 21,503 (18.5% of reference genes in cotton) genes were detected in the meiosis stage of WT and mutant anthers, respectively; 37,352 (32.1% of reference genes in cotton) and 28,833 (24.7% of reference genes in cotton) genes were detected in the tetrad stage of WT and mutant anthers, respectively; and 28,066 (24.1% of reference genes in cotton) and 25,936 (22.3% of reference genes in cotton) genes were detected in the uninucleate microspore stage of WT and mutant anthers, respectively (Figure 3a). Each stage of WT and mutant anthers express more than 20,000 genes, consistent with results from other plants in that 20,000–30,000 genes are expressed in anthers [26,27] and anthers express more genes than other organs [28]. Comparing to 6,675 genes identified in cotton leaf development [29], the genes detected in cotton anther development were significantly more than the genes participated in leaf development, confirming the consensus of previous studies and further suggesting the deep sequencing results of cotton anthers were reliable.

The fertile anthers exhibit transcript complexity during three anther stages: of a total of 42,488 genes that were expressed over three stages of WT anthers, 22,131 were constitutively expressed, 13,269 were stage-specific and 7,088 were expressed at two stages (Figure 3b; see Additional file 4 for the gene list for each category). These dynamic patterns of gene expression reinforce the conclusion that male reproductive development is a highly complex process in plants. Strikingly, during the three stages of WT anthers, the transcriptome in the tetrad stage were significantly more than other two stages. However, the transcriptome in the tetrad stage is lower than the same two stages in maize anther development [27]. This reversely increased transcript diversity in the tetrad stage of cotton anthers indicates that cotton anther development may have some unique gene regulation processes and pathways.

During the transition from the meiosis to tetrad stage, there was a major transcriptome change: 10,022 stage-specific transcripts expressed in the tetrad stage anther (fufous checked bar in Figure 3d, see Additional file 4) and 3,764 transcripts expressed in the meiosis stage anther were not detectable at the tetrad stage (green checked bar in Figure 3d, see Additional file 4). Notably, the stage-specific transcripts in the tetrad stage of WT anthers were also significantly more than other two stages (Figure 3b), indicated that the anther in the tetrad stage might express a distinctive suite of genes for core cellular functions, which may be vital for tetrad generation or microspore separation during the cotton anther development. During the subsequent transition from the tetrad to uninucleate microspore stage, 1,372 transcripts were specific (fufous checked bar in Figure 3d), while 2,525 transcripts (blue bar in Figure 3d, see Additional file 4) that were present at both the meiosis and tetrad microspore stages were no longer detectable.

Although mutant anthers were defective, the transcriptome of mutant anther was also highly dynamic. Of a total of 34,932 genes that are expressed over the three stages of mutant anthers, 16,145 transcripts were shared across all the three mutant anther stages, 9,737 were stage-specific transcripts and 9,050 genes were expressed at two stages (Figure 3c, see Additional file 5). During the meiosis to tetrad stage transition, 4,376 new transcripts appeared (fufous checked bar in Figure 3d) and 3,794 transcripts that were expressed in the meiosis stage anther were not detectable at the tetrad stage (green checked bar in Figure 3d, see Additional file 5). At the subsequent tetrad to uninucleate microspore transition, 2,305 transcripts appeared (fufous checked bar in Figure 3d), and 1,564 transcripts (blue bar in Figure 3d, see Additional file 5) present at both the meiosis and tetrad microspore stages were no longer detectable.

In comparison with the three stages of WT anthers, transcripts have differentially degrees of reduction in three corresponding stages of mutant anthers (Figure 3a). Especially, the number of stage-specific transcripts decreased more than twofold in the tetrad stage of mutant anthers (10,022 and 4,627 stage-specific transcripts detected in the tetrad stage of WT and mutant anthers, Figure 3b and c). Considering the observations of tapetal cells underwent acute and abnormal degradation in the tetrad stage of mutant anthers, so many stage-specific transcripts missing in the tetrad stage of mutant anthers were likely to disrupt the temporally coordinated growth and differentiation of anther cells, resulting in the observed defects in tapetal cell division.

**Figure 4:**
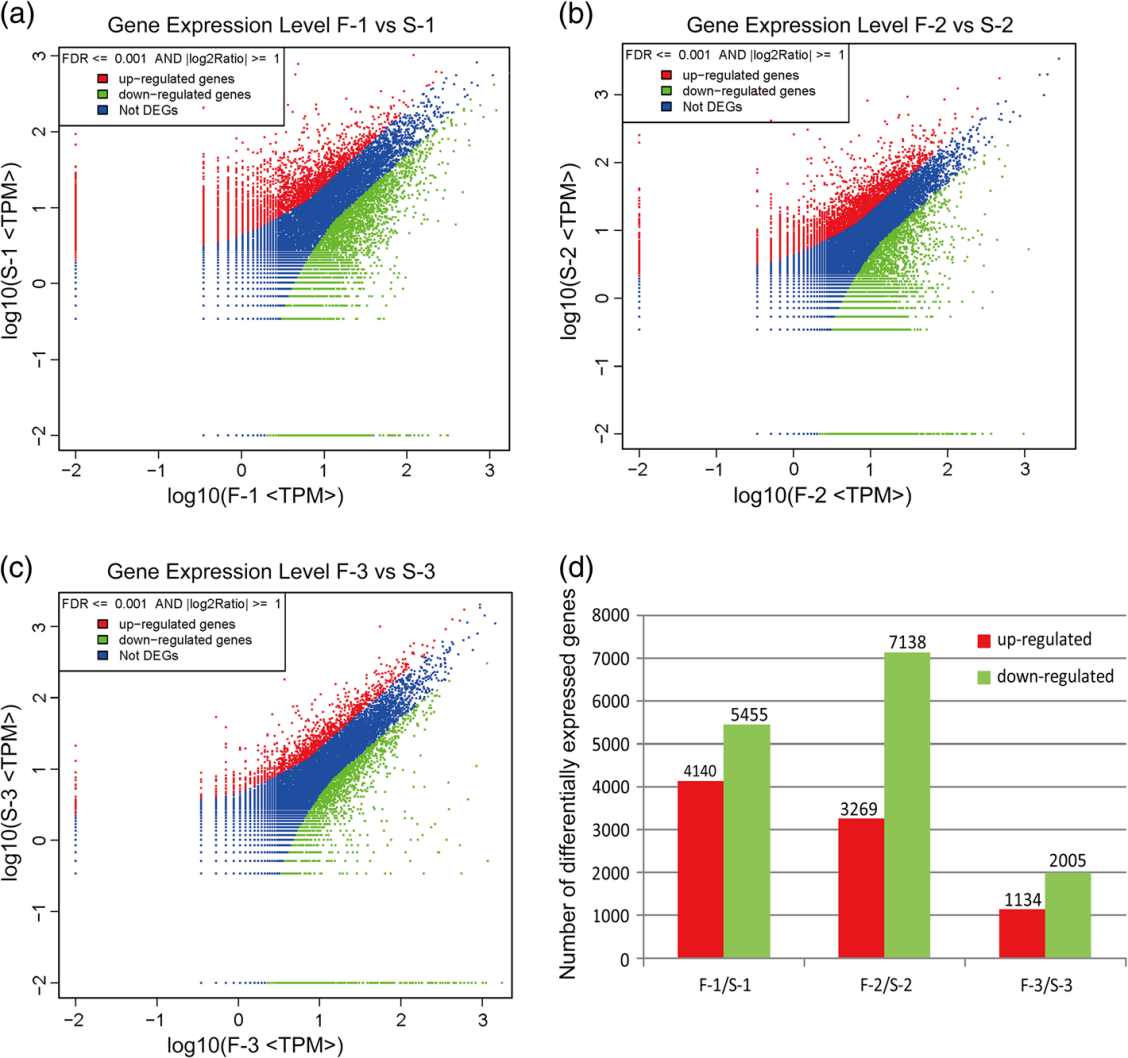
**Differentially expressed genes across all libraries.** All genes mapped to the reference sequence were examined for differences in their expression across the different libraries. (**a**), (**b**) and (**c**) Genes expression levels at meiosis, tetrad and uninucleate microspore stages of the WT and GMS mutant anthers, respectively. (**d**) The number of differentially expressed genes at three stages of WT and mutant anthers. F-1 and S-1: meiosis stage of WT and GMS mutant anthers; F-2 and S-2: tetrad stage of WT and GMS mutant anthers; F-3 and S-3: uninucleate microspore stage of WT and GMS mutant anthers.

To compare the differential expression genes at same developmental stages, the number of raw clean tags in each library was normalized to the number of transcripts per million (TPM) clean tags to obtain the normalized gene expression level, then differentially expressed genes between the WT and GMS mutant samples were identified by an algorithm developed by Audic et al. [30]. In total, 9,595 genes were differentially expressed at the meiosis stage of WT and GMS mutant anther (Figure 4d, see Additional file 6), representing the reprogramming of 30% of the normal transcriptome (9,595/32,120). Among these genes, 4,140 (43%) were up-regulated and 5,455 (57%) were down-regulated at the meiosis stage of GMS mutant anthers (Figure 4d, see Additional file 6). The significant impact of the GMS mutant anther development persisted through the subsequent two stages with 10,407 (25%, 10,407/41,365) genes differentially regulated at the tetrad stage, and 3,139 (10%, 3,169/31,465) genes differentially regulated at the uninucleate microspore stage (Figure 4d, see Additional file 6). Most of the genes were also down-regulated at subsequent stages of GMS mutant anther development, i.e. 7138 (69%) and 2005 (64%) genes at the tetrad and uninucleate microspore stages, respectively (Figure 4d). These results showed that down-regulated genes outnumbered up-regulated genes at the three stages of GMS mutant anther development, which indicated that expression of the majority of genes was suppressed in GMS mutant anther development. Especially at the tetrad stage in the GMS mutant anther, down-regulated genes outnumbered up-regulated genes by more than two-fold, this fact was consistent with the observation that the tapetum degradation mainly occur at the tetrad stage in the GMS mutant anther.

### Genes associated with male sterility in GMS mutant anthers

To characterize the functional consequences of gene expression changes associated with male sterility, a pathway analysis of the differentially expressed genes based on the KEGG database was performed. The results indicated that several key branch-point genes that participate in histone modification and DNA methylation, hormone signaling, sucrose and starch metabolism, the pentose phosphate pathway, glycolysis, flavonoid metabolism, programmed cell death (PCD), cell wall loosening, and reactive oxygen species (ROS) generation/scavenging, were differentially expressed in GMS mutant anthers (Additional file 7).

In the present study, we detected two methyltransferases and three histone constitution-related genes were differential expressed between the WT and GMS mutant anther development. These genes included those that encode histone H3, histone H2B, 24-sterol C-methyltransferase, and gamma-tocopherol methyltransferase. Three histone constitution-related genes were distinctly up-regulated at the tetrad stage in GMS mutant anthers, but exhibited lower expression at the uninucleate microspore stage in GMS mutant anthers (Table 2). Two methyltransferase genes were gradually down-regulated in three anther developmental stages of WT, but they were gradually up-regulated in three anther developmental stages of the GMS mutant (Table 2).

**Table 2:** Selected genes with altered expression in anthers of the GMS mutant

**Functional**	**Unigene**	**Gene annotation**	**TPM (transcript per million clean tag)**					
**group**	**accession**		**F-1**	**F-2**	**F-3**	**S-1**	**S-2**	**S-3**
Histone modification and DNA methylation								
	TC179702	24-sterol C-methyltransferase	114.28	88.21	84.44	32.82	82.49	183.09
	TC179697	Gamma-tocopherol methyltransferase	72.48	43.18	32.48	37.44	51.22	79.54
	TC208258	Histone H3	12.66	2.36	4.56	0	16.43	1.87
	DT048644	Histone H2B	38.15	4.22	20.72	8.89	32.32	8.18
	TC201522	Histone H2B	26.7	0.67	5.97	9.74	21.73	4.09
Hormone/signaling								
	TC202864	Gibberellic acid receptor	6.59	3.88	5.97	5.98	10.24	2.04
	TC194111	Gibberellin 3-hydroxylase 1	27.57	2.53	6.85	0	17.84	1.7
	DW512177	DELLA protein	11.97	3.54	5.09	0	7.24	3.75
	TC212596	Ethylene receptor	14.56	9.16	18.78	7.18	18.19	18.22
	TC179902	Ethylene-responsive transcription factor	16.65	3.88	12.99	15.04	17.49	12.09
	BF269053	ACC oxidase 4	144.97	76.41	132.3	47.7	172.2	195.01
	TC183353	S-adenosylmethionine synthetase	105.2	46.72	98.31	40.01	42.39	167.7
Sucrose and starch metabolism								
	TC218640	Sucrose transporter 1	3.12	0	114.2	5.13	3.71	0.68
	TC199514	Sucrose synthase	33.81	15.52	82.86	12.99	27.73	41.9
	BE055698	Sugar transferase	2.25	13.16	10.53	69.58	1.41	12.26
	TC206300	Isoamylase	4.51	10.63	8.95	7.69	4.24	5.79
	ES824058	alpha-1,4-glucan phosphorylase L isozyme	0.69	13.83	0.53	0	0.71	0
	TC216914	Glycosyl hydrolase	0.52	14.51	0.35	6.33	0.35	0
	TC200529	Granule bound starch synthase	1.04	15.86	1.58	2.74	3.89	1.7
	TC217124	Hexose transporter 6	11.44	8.1	12.29	2.56	12.01	7.32
	TC182973	Starch branching enzyme II-1	7.63	7.25	37.74	9.23	5.83	8.52
Pentose phosphate pathway								
	TC217284	Fructose bisphosphate aldolase	3.47	0.51	4.92	2.91	2.83	0.34
	TC197807	6-Phosphogluconate dehydrogenase	14.57	83.66	21.94	9.92	24.9	12.94
Glycolysis								
	TC218576	Hexokinase	10.92	7.59	13.34	0	10.77	1.7
	CF075621	Phosphofructo kinase	13.53	14	27.56	17.61	20.67	8.69
	TC186567	Phosphoglycerate kinase	24.1	7.76	15.45	0	28.79	4.09
	TC213442	Pyruvate kinase	45.78	49.25	29.14	47.53	23.14	62.68
	TC187515	Alcohol dehydrogenase	0.52	3.04	0.88	0	1.59	0.34
Flavonoid metabolism								
	TC222327	Chalcone synthase	27.87	4.89	14.22	4.51	8.13	12.26
	TC198373	Flavonoid 3′,5′-hydroxylase	37.69	15.19	18.78	27.35	21.93	14.82
	TC180742	Anthocyanidin reductase	599.13	429.3	1736	190.4	499.6	597.7
	TC185652	Leucoanthocyanidin reductase 1	51.12	7.93	67.24	25.66	17.84	52.97
Program cell death (PCD)								
	TC216464	Cysteine proteinase inhibitor	2.6	17.04	66.53	0	9.54	60.46
	TC201153	Cysteine proteinase	4.51	6.58	2.63	5.98	4.24	4.6
	TC184452	Cytochrome C	15.9	15.01	31.6	32.08	16.07	65.06
Cell wall development								
	TC195867	Alpha-tubulin	14.05	7.93	21.59	5.98	14.48	17.54
	TC212355	Beta-galactosidase	0	0	0	15.95	133.42	304.23
	TC224657	Katanin-like protein	19.6	6.24	8.78	23.93	20.67	12.09
	TC214470	Actin depolymerizing factor 1	3.59	7.93	8.08	13.3	8.13	14.31
	DN827899	Actin depolymerizing factor 2	7.52	53.34	79.03	119.48	63.42	143.78
Reactive oxygen species (ROS) generation/scavenging								
	TC197377	Peroxidase precursor	6.94	1.69	5.09	0	4.06	3.24
	ES840062	Class III peroxidase	1.56	3.2	2.98	15.9	0.71	1.87
	TC203760	Cytosolic ascorbate peroxidase 1	3.12	9.11	1.23	5.3	2.83	1.02
	TC189625	Peroxiredoxin	13.18	3.71	15.1	6.33	12.89	14.31
	TC179896	Superoxide dismutase	70.58	71.35	88.83	6.15	43.63	74.09
	TC206683	Catalase isozyme 1	106.99	56.17	275.2	83.09	222.2	106.9
	TC180628	Glutathione S-transferase	1.21	0	0.35	0	13.78	2.55

Several genes implicated in ethylene synthesis and GA signaling, the expression of which changed significantly in the GMS mutant anther, were identified. These differentially expressed genes included those that encode an ethylene receptor, ethylene-responsive transcription factor, ACC oxidase 4, and GA receptor. These genes were also distinctly up-regulated at the tetrad stage in GMS mutant anthers, but exhibited lower expression at the uninucleate microspore stage in GMS mutant anthers (Table 2).

Changes in expression of carbon synthesis genes were apparent in the GMS mutant anthers. Starch and sucrose production declined as transcript levels of the starch-branching enzyme and sucrose synthetase decreased. Most genes that participated in starch and sucrose metabolism showed much lower expression at the meiosis and uninucleate microspore stages, but were up-regulated at the tetrad stage in GMS mutant anthers. Notably, we detected much higher expression of sugar transferase at two anther developmental stages in the GMS mutant, especially at the meiosis stage, and expression of sugar transferase was twenty-fold higher than that at the same stages of WT anther development. These findings indicated that decreased synthesis and transporting of sucrose and starch occurred in GMS mutant anthers.

Similar to most of the genes involved in starch and sucrose metabolism, many genes associated with glycolysis and the pentose phosphate pathway were up-regulated at the tetrad stage, but down-regulated at the meiosis and uninucleate microspore stages, of GMS mutant anthers. These genes included those that encoded hexokinase, phosphofructokinase, phosphoglycerate kinase, pyruvate kinase, and alcohol dehydrogenase, as well as genes that encoded fructose-bisphosphate aldolase and 6-phosphogluconate dehydrogenase.

**Figure 5:**
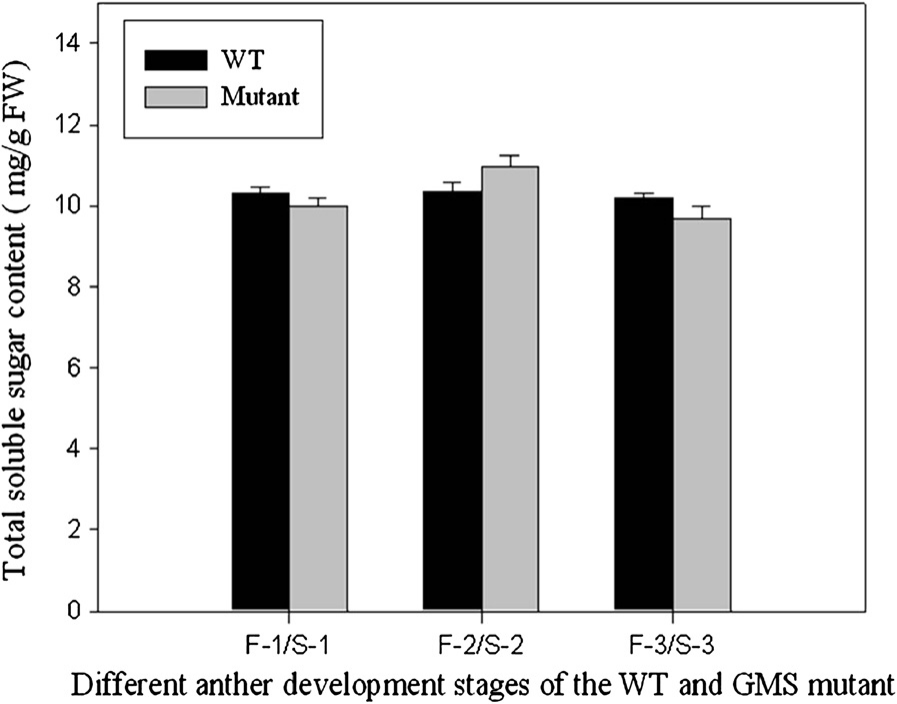
**Total soluble sugar content in WT and GMS mutant anthers.** Data represent the mean and standard error from three replications. F-1 and S-1: meiosis stage of WT and GMS mutant anthers; F-2 and S-2: tetrad stage of WT and GMS mutant anthers; F-3 and S-3: uninucleate microspore stage of WT and GMS mutant anthers. FW: Fresh weight.

Consistent with these changes in transcript levels, the total soluble sugar content showed a slight, but non-significant, decrease at the meiosis and uninucleate microspore stages in GMS mutant anthers (Figure 5), which demonstrated that sugar supply was decreased at the meiosis and uninucleate microspore stages of GMS mutant anther development. Interestingly, the decreased sucrose and starch synthesis, and decreased glycolysis and pentose phosphate metabolism, were consist with the shortage of nutrient substances at uninucleate microspore stag of GMS mutant anther, which indicated that carbon and energy metabolism was suppressed at advanced anther developmental stages in the GMS mutant.

Genes involved in flavonoid metabolism also have the same expression patterns in the GMS mutant anthers, such as chalcone synthase and flavonoid 3′, 5′-hydroxylase, anthocyanidin reductase, and leucoanthocyanidin reductase were down-regulated at the meiosis and uninucleate microspore stages, but were up-regulated at the tetrad stage, in GMS mutant anthers (Table 2). However, genes involved in cell wall loosening, such as a beta-galactosidase, katanin-like protein, actin depolymerizing factor 1, and actin depolymerizing factor 2, were up-regulated at the three developmental stages in the GMS mutant anthers (Table 2). These observations indicated that the actin cytoskeleton balance may be disturbed in the GMS mutant anther, which would directly affect pollen cell wall development.

In active oxygen metabolism and PCD pathways, expression of several ROS generation/scavenging-related genes and PCD-related genes were changed in the GMS mutant anthers. Up-regulated genes included those that encode cysteine proteinase and cytochrome *c*, and down-regulated genes included those that encode a cysteine proteinase inhibitor, peroxidase precursor, class III peroxidise, Cu/Zn superoxide dismutase (SOD), and catalase isozyme, at the uninucleate microspore stage of GMS mutant anthers (Table 2).

### Tag-mapped genes were confirmed by qRT-PCR

**Figure 6:**
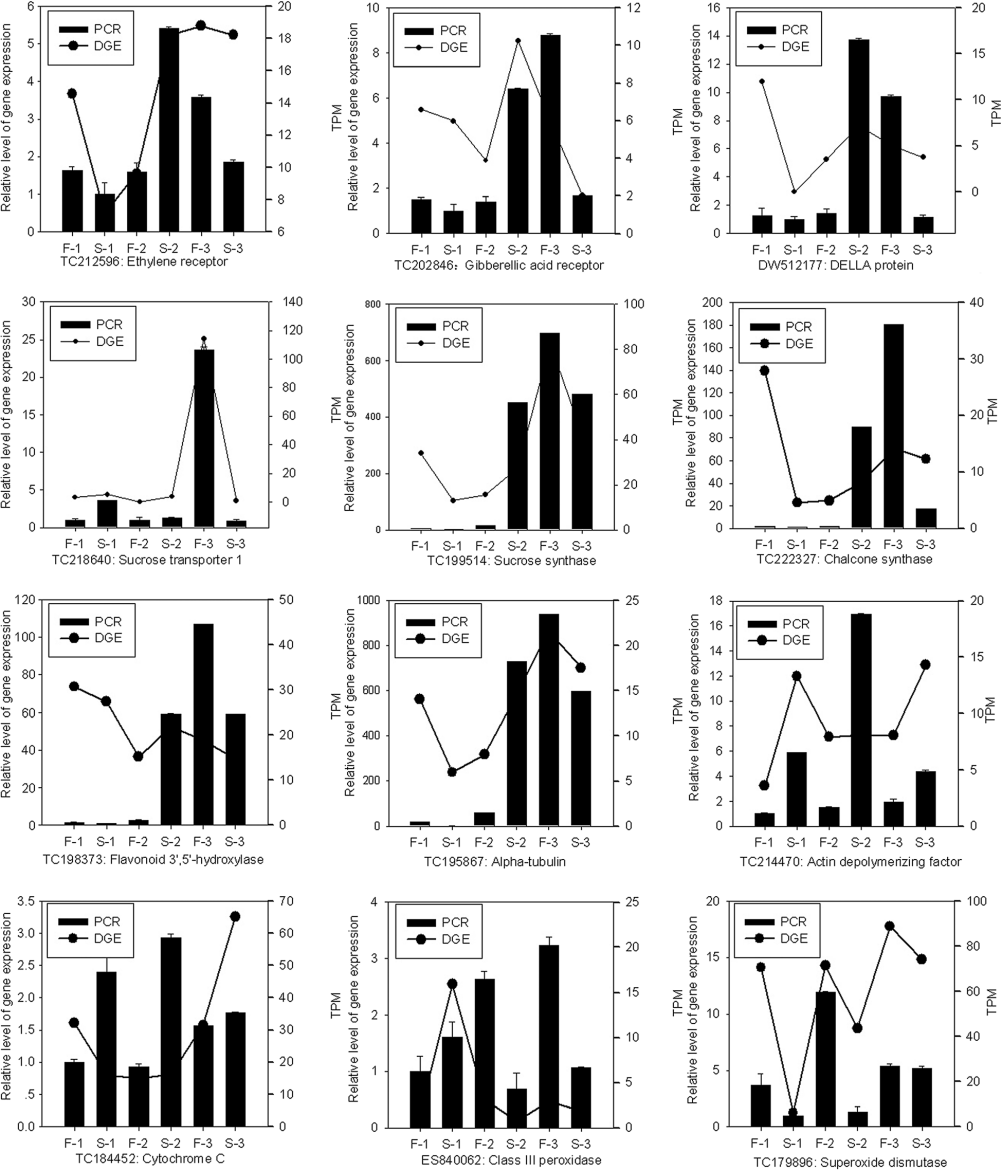
**Quantitative RT-PCR validation of tag-mapped genes associated with cotton anther development.** TPM, Transcription per million mapped reads. F-1 and S-1: meiosis stage of WT and GMS mutant anthers; F-2 and S-2: tetrad stage of WT and GMS mutant anthers; F-3 and S-3: uninucleate microspore stage of WT and GMS mutant anthers. Relative expression levels were calculated using *18S* RNA as an internal control.

To confirm the tag-mapped genes in the WT and GMS mutant anthers, 12 genes were selected for qRT-PCR analysis at the three anther developmental stages. Representative genes selected for the analysis were those involved in hormone signaling, glycolysis, flavonoid metabolism, sucrose and starch metabolism, and active oxygen metabolism pathways because several phenomena accompany manifestation of male sterility, such as nutrient substance shortage, ATP depletion, and loss of cell wall and membrane integrity [31]. The expression of the 11 genes (ethylene receptor, GA receptor, DELLA protein, sucrose transporter 1, sucrose synthase, chalcone synthase, flavonoid 3′, 5′-hydroxylase, alpha-tubulin, actin depolymerizing factor, class III peroxidase, and SOD) indicated by qRT-PCR agreed well with the tag-sequencing analysis patterns (Figure 6). Only one gene (cytochrome *c*) did not show consistent expression between the qRT-PCR and tag-sequencing data sets.

## Discussion

In flowering plants, the production of functional pollen grains is a prerequisite for the propagation, which relies on cooperative functional interactions between gametophytic and sporophytic tissues within the anther. As a non-photosynthetic male reproductive organ, anther must obtain photosynthetic assimilates predominantly from source organs to support pollen development and maturation [32]. The sink strength of anthers is highest at early stages of flower development, with the anthers requiring large amounts of sugar to support their development [33]. At advanced stages, pollen maturation requires the accumulation of starch, which is converted from sugar and functions as an energy reserve for germination [34]; thus, the presence of sufficient levels of sucrose is of vital importance for growth of the male reproductive cells in plants. Disturbances in sugar metabolism and unloading in the anther can significantly impair pollen development and cause male sterility [35].

To ensure reproductive success, flowering plants established two barriers for protecting the pollen grains from various environmental stresses, such as bacterial and fungal attacks. One barrier is the anther wall, which covered by a cuticle can protect the anther from various stresses. Another barrier is the cell wall of the pollen grain, which can be divided into three layers: an outer extine, an inner intine, and a lipid- and protein-rich pollen coat in the crevices of the extine. The extine is a multi-layered structure, primarily consisting of sporopollenin [36]. Sporopollenin is a complex polymer composed of fatty acids and phenolic compounds [37]. The sporopollenin polymer is responsible for the extine’s unparalleled physical strength, chemical inertness, and elasticity to protect pollen against various stresses. After anther dehiscence, the extine forms the outer wall of the pollen grain. These two major barriers are vital for the development of viable pollen grains and male sterility [38].

More importantly, anther development is apparently influenced by ethylene and gibberellic acid (GA). Ethylene is a key gaseous phytohormone that regulates many aspects of plant growth and development [39,40]. The mature pollen is characterized by a high content of ethylene. During the anther development, fertile male gametophyte development is accompanied by two peaks of ethylene production by anther tissues [41]. The first peak occurs during microspore development simultaneously with degeneration of both tapetal tissues and the middle layers of the anther wall. The second peak coincides with maturation and dispersal of the pollen grains. GA also plays an essential role in the regulation of reproductive development, especially of anther development. In *A*. *thaliana*, GAs can accelerate flowering, even under short-day conditions. In maize (*Zea mays*) and castor bean (*Ricinus communis*), GAs can promote the development of female flowers [42,43]. Conversely, GA deficiency or insensitivity in several plant species causes abnormal development of anthers, which often leads to male sterility [44].

To explore the key underlying molecular switches resulting in the male sterility of the cotton GMS mutant, six DGE libraries were set up during the anther development of Dong A WT and GMS mutant in the current work. To the best of our knowledge, the present study is the first to attempt to perform a deep sequencing of the Dong A WT and GMS mutant in cotton anther development, which may facilitate identification of systemic gene expressions and regulatory mechanisms for male sterility.

After analyzing the differences in gene expressions of the WT and the GMS mutant during the anther development, several opposite gene expression were discovered to exist in the anther development of GMS mutant. These genes involved in histone modification and DNA methylation, carbon and energy metabolism, hormone signaling, pollen cell wall development, and ROS metabolism. For example, three histone constitution-related genes and two methyltransferase genes were differentially expressed in the GMS mutant anthers (Table 2). Both histone modification and DNA methylation play essential roles in genome management, and control gene expression or silence [45]. As a main enzyme for DNA methylation, DNA methyltransferase is not only associated with DNA methylation, but also link to many important biological activities, including cell proliferation, senescence [46]. In the present study, three histone constitution-related genes encoding histone H3 and histone H2B were up-regulated at the tetrad stage, but down-regulated at the meiosis and uninucleate microspore stages in the GMS mutant anther. The much lower expression of these histone constitution-related genes at the meiosis and uninucleate microspore stage of GMS mutant anther may affect the normal chromosome structure and influence the functional gene expression in the GMS mutant anther. Besides, two methyltransferase genes were gradually up-regulated in the three stages of GMS mutant anthers, but they were gradually decreased in the three corresponding stages of WT anthers (Table 2), indicating that the expression of these methyltransferase genes were activated in the anther development of GMS mutant, but they were suppressed in the anther development of WT. The increased expression of methyltransferas genes in the GMS mutant anther development may change the level of DNA methylation and repress the functional gene expressions in the anther development of GMS mutant, which may explain the results of the down-regulated genes outnumbered up-regulated genes at the three stages of GMS mutant anther development (Figure 4).

The present research also found that expression of many key branch-point genes associated with the sucrose and starch metabolism, the pentose phosphate pathway, and the glycolysis pathways were affected in GMS mutant anthers. All of these genes with potential roles in carbon and energy metabolism were down-regulated at the uninucleate microspore stage in GMS mutant anthers, which indicated that energy metabolism was disturbed or suppressed during the later anther developmental stage of GMS mutant. In facts, pollen requires the accumulation of sucrose and starch as an energy reserve for maturation in later anther developmental stages. These opposite gene expression patterns in the GMS mutant anthers may directly reduce sucrose transportation and accelerate sucrose depletion in anthers, leading to nutrient substance deficiency in the GMS mutant and related to male sterility.

More interestingly, five genes involved in ethylene metabolism were differential expressed in WT and GMS mutant anthers. Previous studies on male sterility noted changes in ethylene and gibberellic acid (GA) content in GMS lines [47,48]. These genes were up-regulated by almost two-fold at the tetrad stage in the GMS mutant anther, but down-regulated at the meiosis and uninucleate microspore stages in the WT anther. Considering the TEM observations at the tetrad stage in GMS mutant anthers, surprisingly we found that the higher expression of genes involved in ethylene metabolism coincided with the peak in tapetal tissue degeneration in the GMS mutant anther, which indicated that the higher expression of these ethylene metabolism-related genes may directly lead to the premature degeneration of the tapetal layer in GMS mutant anthers. During the uninucleate microspore stage in WT and GMS mutant anthers (the stage of pollen prematuration), these genes showed lower expression in the GMS mutant anther but higher expression in the WT anther. Given that the pollen grains in the GMS mutant are not dispersed, this opposing pattern of ethylene metabolism expression in the GMS mutant anther suggests that ethylene also may act as an important signal mediating the response to tapetal degeneration and pollen maturation.

The present research also found three GA metabolism-related genes (GA receptor, gibberellin 3-hydroxylase 1, and DELLA protein) that were up-regulated at the tetrad stage, but down-regulated at the meiosis and uninucleate microspore stages in the GMS mutant anther (Table 2). Given the essential roles of the GA receptor, and involvement of the DELLA protein in GA signaling for pollen extine production and male sterility in A. thaliana and rice [49,50], the much lower expression of these GA metabolism-related genes at the uninucleate microspore stage of GMS mutant anther development could affect pollen extine formation.

Interestingly, the expression pattern of these GA metabolism-related genes was similar with the expression patterns of the ethylene metabolism-related genes at the same stages of WT and GMS mutant anther development. Ethylene treatment can markedly affect GA content; however, GA does not significantly affect ethylene-related gene expression in relation to regulation of hypocotyl and root length, which suggests that ethylene acts upstream via GA to regulate hypocotyl and root development [51]. On the basis of the similar expression pattern of the ethylene and GA metabolism-related genes in the WT and GMS mutant anthers, we reasoned that ethylene also may act upstream via GA to regulate cotton anther development, thereby leading to the higher expression of GA metabolism-related genes at the tetrad stage, but lower expression at the meiosis and uninucleate microspore stages in GMS mutant anthers. The opposing expression pattern of these key branch-points genes involved in ethylene and GA metabolism during GMS mutant anther development may act as an important hormone signal-mediating response to male sterility.

More importantly, expression of key branch-point genes involved in flavonoid metabolism, which include chalcone synthase, flavonoid 3′, 5′-hydroxylase, anthocyanidin reductase, and leucoanthocyanidin reductase increased at the tetrad stage but were down-regulated at the uninucleate microspore stage in GMS mutant anthers, which indicated that flavonoid metabolism was initially activated, then suppressed at advanced stages of GMS mutant anther development (Figure 6). In particular, at the uninucleate microspore stage in GMS anthers, the expression of chalcone synthase and flavonoid 3′, 5′-hydroxylase was down-regulated ten-fold and two-fold, respectively. Flavonoids are important for pollen germination and fertility in several plant species, but not for *A*. *thaliana*[52]. Chalcone synthase and flavonoid 3′, 5′-hydroxylase are two key branch-point genes for flavonoid biosynthesis, which is vital important for pollen extine formation [53]. Mutation in the key branch-point genes involved in flavonoid metabolism in the former plant species results in male sterility and the absence of flavonoids in the mature stamens. Exogenous application of flavonols can completely restore their fertility [54]. The much lower expression of these key branch-point genes may lead to the excessive reduction of flavonoid synthesis and accumulation of phenolic compounds at the uninucleate microspore stage in GMS mutant anthers, which are considered to be vital for extine formation and related to male sterility in the GMS mutant.

The present research also found that several genes involved in pollen cell wall development were affected in the GMS mutant anther development. For example, at the uninucleate microspore stage, lower expression of the alpha-tubulin and tubulin alpha-2 chain genes in GMS mutant anthers was detected. Reduction of the tubulin level could affect tube elongation and result in aberrant cytoskeletal structures in microspores [55]. In thermo-sensitive genetic male sterile line of wheat, anthers show a shrunken plasma membrane and aberrant actin cytoskeletal structures, which indicate that the cytoskeletal organization is altered during anther development [56]. In contrast, actin depolymerizing factor genes were highly expressed at the three anther developmental stages in the GMS mutant (Figure 6). In *G*. *hirsutum*, over-expression of the actin depolymerizing factor (GhADF7) can alter the balance of actin depolymerization and polymerization, thus leading to defective cytokinesis and partial male sterility [57]. Surprisingly, in the present study beta-D-glucosidase (an important cell wall-degrading enzyme) exhibited higher expression at the meiosis and uninucleate microspore stages in the GMS mutant anthers. The abnormal expression of alpha-tubulin, tubulin alpha-2 chain, actin depolymerizing factor, and beta-D-glucosidase genes in the GMS mutant anther, may directly influence the synthesis of F-actin cytoskeleton and further alter the cell wall developmental pattern in the GMS mutant anther development.

Notably, compared with the WT anther, much lower expression of the peroxidase precursor, class III peroxidise, Cu/Zn SOD, and catalase isozyme1 was detected at the uninucleate microspore stage in the GMS mutant anther, which indicated that not only various ROS-scavenging enzymes were suppressed, but also their generation may be blocked in the GMS mutant anther. In plants ROS have prominent roles in the induction, signaling, and execution of PCD [58]. In the cotton CMS line, at the peak of anther abortion, excessive accumulation of ROS and a significant down-regulation of ROS-scavenging enzyme expression coincide with male cell death in male sterility [59]. In present study, the decreased ROS-scavenging enzyme activity in the GMS mutant anther may lead to a transient oxidative burst and significantly increased ROS accumulation at the uninucleate microspore stage in the GMS mutant anther. Furthermore, at the uninucleate microspore stage (the peak stage of anther abortion in the GMS mutant anther), we detected two PCD-related genes (cysteine proteinase and cytochrome *c*) that were up-regulated in the GMS mutant anther. It is considered that ROS can trigger and promote the expression of cysteine proteinase and cytochrome *c*, which lead to PCD in plants [60]. These results are consistent with recent findings that male cell death is coincides with excessive formation of ROS at the peak of anther abortion in a cotton CMS line. Surprisingly, a cysteine proteinase inhibitor is down-regulated at the three stages of GMS mutant anther development. On the basis of these results, it is possible that the expression balance of the cysteine proteinase and cysteine proteinase inhibitor might be disturbed in later GMS mutant anther development, thus resulting in the imbalance in oxidative metabolism and male cell death in the GMS mutant.

## Conclusion

The present results indicate that expression of genes participated in many diverse molecular functions were altered during anther development in the GMS mutant. Several key branch-point genes involved in histone modification and DNA methylation, hormone signaling, carbon and energy metabolism, pollen wall development, and ROS generation or scavenging were differentially expressed in the GMS mutant anther. To some degree, compared to the WT anther at the same developmental stage, these differentially expressed genes exhibit opposite expression patterns in the GMS mutant anther, which indicates that the hormone signals and energy metabolism are disturbed or blocked during anther development in the GMS mutant. These changes may be the major factors that related to male sterility. These findings provide systemic insights into the mechanism of male sterility and contribute to an improved understanding of the molecular mechanism of male sterility in cotton. Some key branch-point genes involved in cotton anther development are good candidate genes for functional analysis of anther development in the future.

## Methods

### Plant materials

Plants of upland cotton (*G*. *hirsutum*) cv. ‘Dong A’ (WT) and the GMS mutant in the ‘Dong A’ background were grown in an experimental field at the China Agricultural Academy of Science Cotton Research Institute under standard field conditions during the spring and summer of 2010. Previous study revealed that when the longitudinal length of buds reach 5.0 mm, 6.5 mm, and 9 mm, respectively, the pollen mother cell of the GMS mutant enter the meiosis, tetrad and uninucleate stages [61]. According to these sampling criterions and combined with microscopic examination of pollen mother cells, developing anthers at these three stages of development were harvested during early morning on the basis of floral bud length. The excised anthers were frozen in liquid nitrogen and stored at −70°C prior to examination. In addition, WT and GMS mutant anthers at the meiosis, tetrad, and uninucleate stages were harvested for total RNA isolation.

### Total sugar content measurement

Anthers were harvested and frozen at −70°C. The samples were ground into a powder with a pestle and mortar. Twenty milliliters of water were added to glass tubes containing 1 g of ground anther tissue, incubated at 100°C for 10 min, then centrifuged at 2500 g for 5 min. A total of 2 mL of a solution containing glucose, fructose, or galactose was prepared. To this solution, 2 mL of a glucose solution was added to achieve final concentrations of 0%, 2%, 4%, 6%, 8%, and 10% glucose for optimization. An anthrone colorimetric method was adopted to determine the total sugar content in the WT and GMS mutant anthers [62].

### Sequencing and library construction

Total RNA was extracted from anthers using the pBiozol Total RNA Extraction Reagent (BioFlux) in accordance with the manufacturer’s instructions. RNA was precipitated with ethanol, dissolved in diethypyrocarbonate-treated water (DEPC) and stored at −70°C. All RNA samples were examined for protein contamination (as indicated by the A_260_/A_280_ ratio) and reagent contamination (indicated by the A_260_/A_230_ ratio) with a Nanodrop ND 1000 spectrophotometer (NanoDrop, Wilmington, DE).

Total RNA purity and degradation were checked with a 1% agarose gel before proceeding. The samples for transcriptomic analysis were prepared using the Illumina kit following the manufacturer’s recommendations. The extracted total RNAs were resolved on a denatured 15% polyacrylamide gel. Briefly, mRNA was purified from 6 μg total RNA using oligo (dT) magnetic beads. Following purification, the mRNA was fragmented into small pieces using divalent cations under an elevated temperature and the cleaved RNA fragments were used for first-strand cDNA synthesis using reverse transcriptase and random primers.

### Quantitative RT-PCR analysis

Quantitative RT-PCR analysis was used to verify the DGE results. The RNA samples used for the qRT-PCR assays were identical to those used for the DGE experiments. Gene-specific primers were designed on the basis of the reference unigene sequences with Primer Premier 5.0 (see Additional file 8). The qRT-PCR assay was performed in accordance with the manufacturer’s specifications. The reactions were incubated in a 96-well plate at 95°C for 10 min, followed by 40 cycles of 95°C for 15 s and 60°C for 60 s. The cotton *18S* RNA gene (forward primer: 5′-ATCAGCTCGCGTTGACTACGT-3′; reverse primer: 5′- ACACTTCACCGGACCATTCAAT-3′) was used to normalize the amount of gene-specific RT-PCR products, and the relative expression levels of genes were calculated with the 2^−ΔΔCt^ method.

## Competing interests

The authors declare that they have no competing interests

## Authors’ contributions

SXY and SLF designed the experiments. SLF performed the field cultivation of cotton plants and anther collection. MZS conceived the study, participated in its design, and drafted and amended the manuscript. MMW performed the experiments. All authors read and approved the final manuscript.

## Supplementary Material

Additional file 1 Distribution of distinct clean tags in six libraries. Click here for file

Additional file 2 Sequencing depth in six DGE libraries. Click here for file

Additional file 3 Mapping of distinct clean tags in the six DGE libraries. Click here for file

Additional file 4 The gene list for each category in three stages of WT anthers. Click here for file

Additional file 5 The gene list for each category in three stages of mutant anthers. Click here for file

Additional file 6 Satistics of differential expressed genes in the six DGE libiaries. Click here for file

Additional file 7 The significant differential expressed genes in the six DGE libiaries. Click here for file

Additional file 8 Primers of selected genes. Click here for file
