# Supplementary figures and images for: Transcriptomic analysis of differentially expressed genes during anther development in genetic male sterile and wild type cotton by digital gene-expression profiling
Source: BMC Genomics. 2013 Feb 12;14:97. doi: 10.1186/1471-2164-14-97 (PMC3599889; doi:10.1186/1471-2164-14-97)

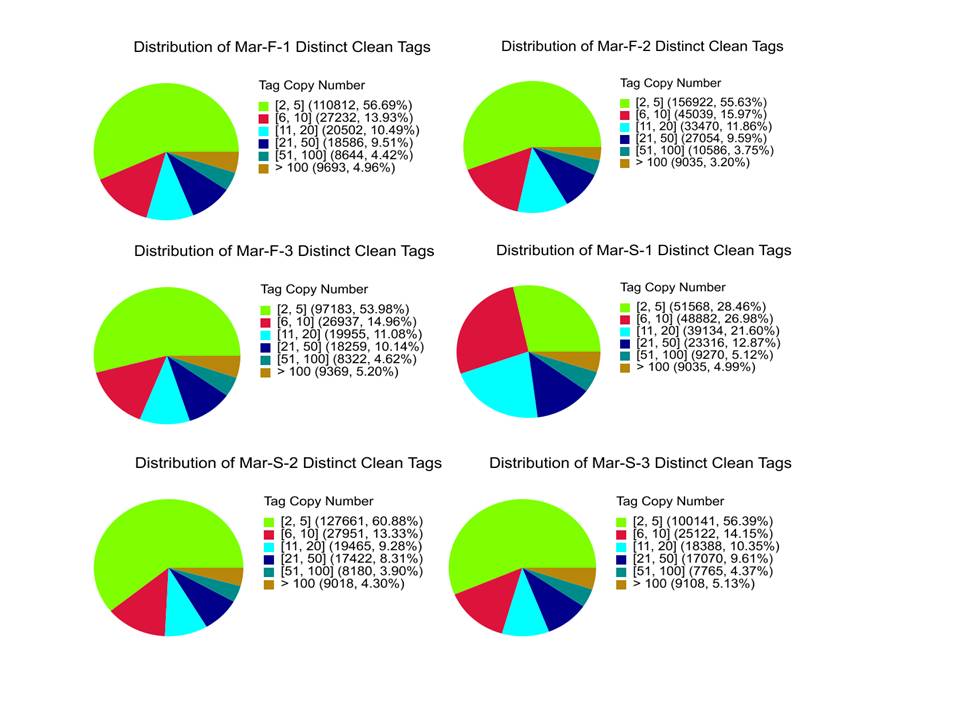

Supplement: Additional file 1 — Distribution of distinct clean tags in six libraries. [file 1471-2164-14-97-S1.jpeg]

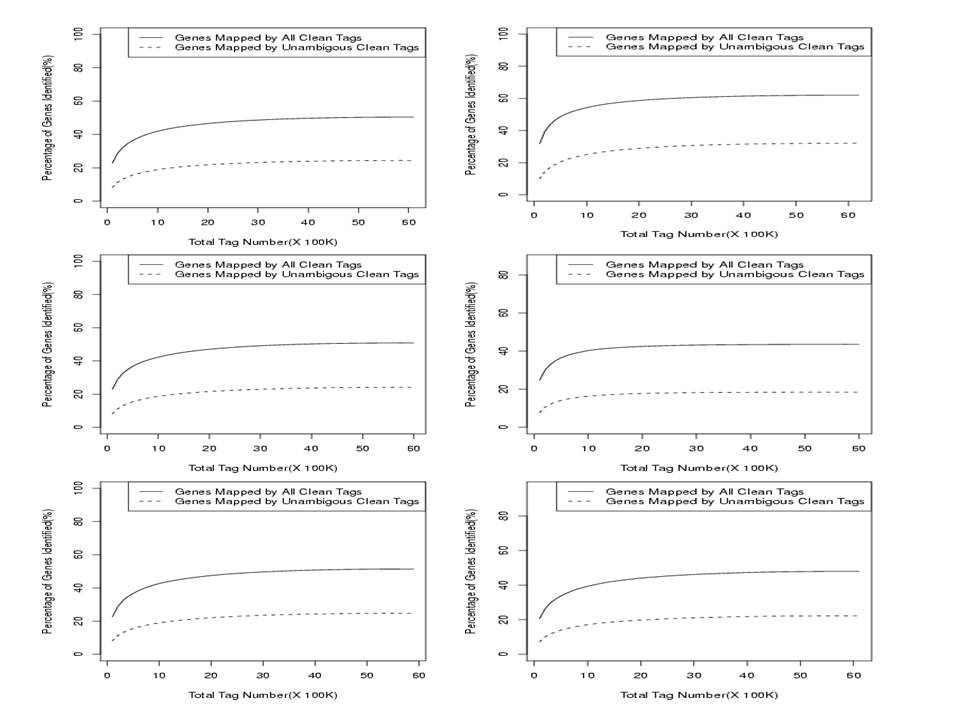

Supplement: Additional file 2 — Sequencing depth in six DGE libraries. [file 1471-2164-14-97-S2.jpeg]

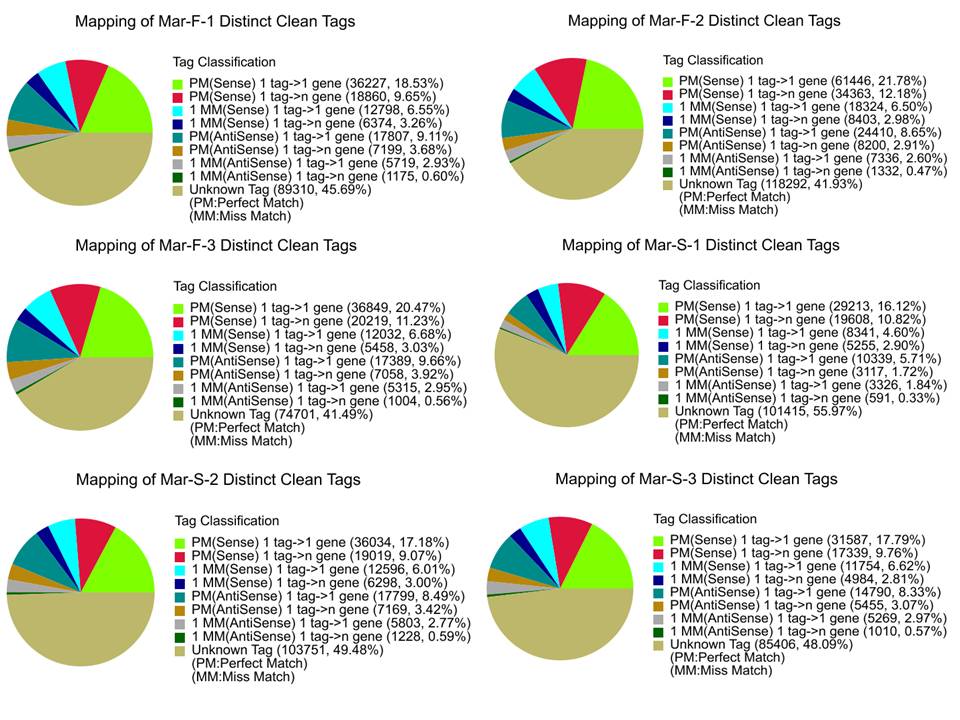

Supplement: Additional file 3 — Mapping of distinct clean tags in the six DGE libraries. [file 1471-2164-14-97-S3.jpeg]
